# Historical Arctic Logbooks Provide Insights into Past Diets and Climatic Responses of Cod

**DOI:** 10.1371/journal.pone.0135418

**Published:** 2015-09-02

**Authors:** Bryony L. Townhill, David Maxwell, Georg H. Engelhard, Stephen D. Simpson, John K. Pinnegar

**Affiliations:** 1 Centre for Environment, Fisheries & Aquaculture Science (Cefas), Lowestoft, Suffolk, United Kingdom; 2 University of Exeter, Biosciences, College of Life and Environmental Sciences, Geoffrey Pope, Stocker Road, Exeter, United Kingdom; Institute of Marine Research, NORWAY

## Abstract

*Gadus morhua* (Atlantic cod) stocks in the Barents Sea are currently at levels not seen since the 1950s. Causes for the population increase last century, and understanding of whether such large numbers will be maintained in the future, are unclear. To explore this, we digitised and interrogated historical cod catch and diet datasets from the Barents Sea. Seventeen years of catch data and 12 years of prey data spanning 1930–1959 cover unexplored spatial and temporal ranges, and importantly capture the end of a previous warm period, when temperatures were similar to those currently being experienced. This study aimed to evaluate cod catch per unit effort and prey frequency in relation to spatial, temporal and environmental variables. There was substantial spatio-temporal heterogeneity in catches through the time series. The highest catches were generally in the 1930s and 1940s, although at some localities more cod were recorded late in the 1950s. Generalized Additive Models showed that environmental, spatial and temporal variables are all valuable descriptors of cod catches, with the highest occurring from 15–45°E longitude and 73–77°N latitude, at bottom temperatures between 2 and 4°C and at depths between 150 and 250 m. Cod diets were highly variable during the study period, with frequent changes in the relative frequencies of different prey species, particularly *Mallotus villosus* (capelin). Environmental variables were particularly good at describing the importance of capelin and *Clupea harengus* (herring) in the diet. These new analyses support existing knowledge about how the ecology of the region is controlled by climatic variability. When viewed in combination with more recent data, these historical relationships will be valuable in forecasting the future of Barents Sea fisheries, and in understanding how environments and ecosystems may respond.

## Introduction

Many studies predict that climate change will modify ecosystems in the future (e.g. [1–3], or have detected impacts of climatic variability on ecosystems in the past [4–6]. However, historical ecological data are often limited in quantity and quality, and can be piecemeal and difficult to access, greatly restricting the power of models for predicting future conditions. Historical data are essential for understanding how environmental factors influence ecosystems over long periods of time and for training models that predict the effects of future climatic changes. Understanding how ecosystems have been modified by past environmental change informs future projections. It is valuable to digitise historical information before it is lost [7] and probe non-standardised historical data to investigate effects of previous climatic influences (e.g. [8].

Due to its inaccessibility the Arctic’s marine systems are relatively understudied compared with other regions such as the North Sea [9–11], although the Barents Sea region has received more attention than much of the Arctic due to Russian and Norwegian fisheries interest. Atlantic cod *Gadus morhua* in the Arctic region around Norway and Russia (also described as Barents Sea, Northeast Arctic and Arcto-Norwegian cod [12], is a major predatory species. Barents Sea cod is the most economically important Barents Sea species, with landings of 432,314 and 438,734 tonnes for Russia and Norway respectively in 2013 [13], from where it is then exported around Europe. Cod stocks in the Barents Sea are currently at high levels not seen since the 1950s [14], when temperatures were similar to those today. Recent studies have attempted to explain how cod and spawning sites are distributed, how such large numbers of fish are sustained, and how available prey resources are exploited (e.g. [15–18]). Scientists have known for over a century that the cod stock in the region can fluctuate greatly [19–20], and research in the early 20^th^ century attributed fluctuations to warming in the Barents Sea and the Arctic (e.g. [20–22]) and changes to currents and hydrography [23]. Researchers appreciated the importance of changing environmental conditions; for example an ICES Special Scientific Meeting assessed how climatic changes relate to fluctuations in northern fish stocks [24], leading to a number of publications focusing on the issue [25–29].

**Fig 1:**
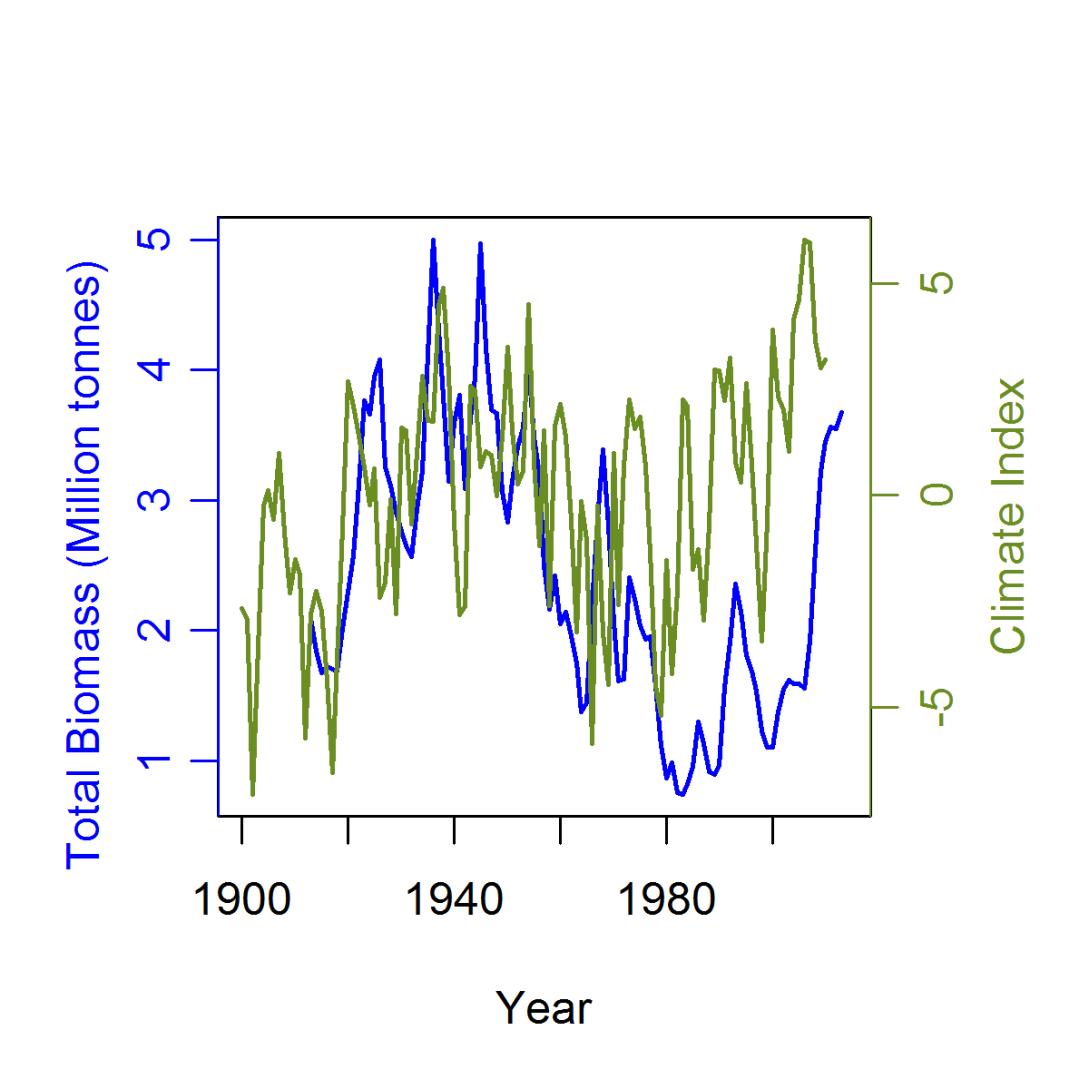
Total cod total stock biomass (blue) in the Barents/Norwegian Sea (ICES sub-areas I and II) from 1916–2013 (1916–1945 from Kjell Nedreaas, IMR, Bergen pers. comm., [40]; 1946–2013 from [13]), compared with a Climate Index (green) for the Barents Sea (1900–2009) [39].

Atlantic cod has become emblematic of climate influences in fish and fisheries [30], and Barents Sea cod are of particular interest, being at the northernmost boundary of the species range at some of the lowest temperatures experienced by the species [31–32]. Temperature directly relates to population size, with a period of warmer temperatures in the Kola section of the south-eastern Barents Sea from 1930–1960 corresponding with high cod catches and biomass, and a cooler period from 1960–2000 corresponding with lower catches and biomass [17, 33]. Barents Sea cod tend to produce stronger year classes in warmer years [34], and there is evidence of a 50% increase in growth rates from the pre-1920s to the 1960s [15, 35]. When warm copepod and euphausiid-rich Atlantic water is brought in from the Norwegian Sea, the warmer temperatures may increase the habitable area for plankton and associated fish stocks, increasing local abundance of cod prey such as capelin *Mallotus villosus* and herring *Clupea harengus* [36]. Following cod spawning, spatial distribution depends on annual ocean currents that carry the larvae and juveniles from spawning grounds along the coast of northern Norway, north and east into the Barents Sea. Current-driven spatial distributions of juvenile cod determine the depths and temperatures experienced later through to the immature stages, thus affecting growth rates and ultimately adult size [37]. In cold years, the size of feeding areas appears to be reduced, with effects on year class strength [38]. Since the 1990s, Kola section annual mean temperatures have increased to ~5°C, and spawning stock biomass (SSB) and total biomass have rapidly risen to levels even higher than the previous warm period. The environmental conditions in the Barents Sea, as illustrated by a Climate Index in Fig 1 based on air temperature, Atlantic water temperature and ice cover [39], correlate closely with these trends in cod biomass [13, 40].

Historical data have proved valuable in assessing past impacts of climate [6, 41–42]. Here, we use newly digitised historical datasets for the Arctic to investigate changes in the distribution of Barents Sea cod, and assess changes in consumption of different prey types. Surveys mainly focused on recording catches of cod and linking catches with environmental variables, but also included stomach content analysis to assess prey that cod and other predators were consuming, and a more general assessment of the distribution of benthic invertebrate species (see [43]). These rescued datasets are particularly valuable because they provide detailed station information alongside the catch and stomach data, in comparison with the more intensive Russian and Norwegian surveys. Russian scientists made initial observations of stomach contents from the 1920s, and collected stomach data from 1930–1960, with at least some of these surveys further south and east of those digitised here [44–45]. Stomach analysis from British expeditions in 1936 and 1937 have been previously published [46] although not the full dataset. Norwegian scientists later began analysing stomach samples and cod catches from 1984 [47] as part of the joint Norway-Russia research programme on trophic relationships in the Barents Sea [45].

This study uses analytical techniques which were not available at the time to consider historical changes in Barents Sea cod catches and diets in the wider context of warm and cool periods. Specifically, we use statistical models to test for: (a) drivers of change in cod catches and prey frequencies; (b) environmental and spatial conditions in which cod catches were higher; (c) periods and times of year at which cod catches were higher; and (d) differences in prey frequencies attributable to environmental, spatial and temporal variables.

## Materials and Methods

During the period 1930–1960, British researchers carried out marine surveys in the Arctic, initially boarding commercial fishing vessels to measure length distribution of cod near Bear Island, in the Svalbard (Spitsbergen) region [48]. No surveys were carried out during World War II, but thereafter the scale of the surveys was increased from 1949 when a dedicated research ship (the RV *Ernest Holt*) was custom-built for the Svalbard-Barents Sea area by the UK Ministry of Agriculture and Fisheries (now part of Defra). Surveys were predominantly carried out in the western Barents Sea and Norwegian Sea, but also included sites around Greenland, Iceland, the Faroe Islands and the North Sea. Surveys have taken place during each of the seasons, but especially during summer and autumn when ice cover in the Barents Sea is lowest.

Cod catch weights, stomach content and hydrographic data were digitised from scientific survey logbooks held in the Centre for Environment, Fisheries & Aquaculture Science (Cefas) archive. Catch weights and hydrographic data were archived in the Cefas Fisheries Survey System (FSS) database, and stomach content data were archived in the DAPSTOM online database [49–50]. Data were checked for quality purposes by mapping sequential sampling stations, plotting the data to identify outliers and unlikely records, and subsequently revisiting logbooks and removing clearly erroneous records if necessary (approximately 0.5% of species records). Sampling campaigns were not always designed in a statistically robust way, but instead the vessels ‘chased’ high cod catches, thus operating in a similar way to commercial vessels. Therefore data stations were not gridded according to a statistically stratified design, rather they were often focussed on areas where high catches were known to be found and thus data gathered was more akin to commercial cod catch per unit effort (CPUE) than to traditional scientific abundance measures. All ships used an otter trawl. Information is very sparse on exact gear design for initial cruises, however after 1949 the *RV Ernest Holt* used a standard otter trawl with a 78 ft (23.7 m) headline, 116ft (35.4 m) groundrope, and 110mm cod end mesh, with and without Vigneron-Dahl gear [48]. Trawl durations were generally two hours. Stomach analyses, although comprehensive, were also not as statistically robust as on current surveys. No data on stomach content mass or stomach fullness were recorded; instead only frequency of occurrence of each prey item or presence data were recorded. In order to convert units of CPUE to modern equivalents it was necessary to transfer catch estimates from numbers of ‘baskets’ or hundred-weight (cwt) and pounds (lbs) to kilograms, assuming that one basket of cod is equivalent to 84 lbs (0.75 cwt) or 38 kg [48], and accepting that there would be variation around this average.

In this study, stations within the Norwegian and Barents Seas (62–80°N 5–55°E) were selected for analyses, with data available for 17 years (1930, 1937, 1938, 1939, 1945, 1948–1959). Environmental parameters available for each station included location, depth, duration of haul, bottom and surface temperature and salinity. Temperature and salinity measurements were not taken at all stations, and bottom salinity (11% of stations) was more commonly recorded compared to surface salinity (0.2% of stations). The mass of the cod catch was used, except where only counts had been recorded (9% of cases), in which case each individual cod was assumed to weigh two kilograms (based on the mean mass of cod where both were recorded), again accepting that there would be variation around this. Cod CPUE in kilograms per hour was then calculated at each station by dividing catch weight by haul duration.

In order to determine the most important variables for explaining cod abundance, the relationship between annual, monthly and daily cod CPUE with longitude, latitude, bottom and surface temperatures, bottom salinity and depth were examined for explanatory trends. These relationships were investigated further using Generalized Additive Models (GAMs) implemented in the *mgcv* package [51] of the statistical software *R* (version 3.0.3; [52]). A GAM determines the interactions between variables using a flexible model that allows for non-linear relationships between several potential predictors and dependent variables using smoothing functions. In this case cod CPUE was the dependent variable in each model and the explanatory variables considered were categorised as either ‘temporal’ (year, month, day of year) or ‘environmental’ (bottom and surface temperatures, bottom salinity and depth), with longitude and latitude included in each case. A third ‘spatio-temporal’ model was also produced using a combination of longitude, latitude and year. The optimal temporal, environmental and spatio-temporal models were chosen by selecting the most suitable distribution through visual assessment of the residual and smooth plots produced by the *mgcv* package, and balancing selection of a high model deviance, a low number of degrees of freedom, and a low UGCV/UBRE score as suggested by guidance [51]. Visual assessment of the plots was essential in model choice to ensure that models had sufficient explanatory power and were not overfitting the data.

The negative binomial distribution family was chosen in the final three models as it allowed for increased variability at larger values of CPUE and dealt more effectively with the high occurrence of zero-values in CPUE data than the other families tested (Gaussian and Tweedie), thus providing a higher explanation of deviance (Table 1). The negative binomial dispersion parameter theta (*θ*) was selected automatically during model fitting. Within *mgcv*, tensor smooth ‘te’ was used for the three-way interaction term within the spatio-temporal model, while the more common ‘s’ smooth was used for one- and two-way interactions in all three models. The smoothing base of ‘ts’ (thin plate regression spline with shrinkage; [51]) was used within the interaction terms for the environmental and spatio-temporal models, as it allows the GAM to smooth with any number of covariates and drops covariates that do not improve the model fit by setting their degrees of freedom to zero. For the interaction between longitude and latitude in the temporal and environmental models, ‘sos’ (splines on a sphere; [53]) was used. This fits latitude and longitude to a sphere and so allows for the narrowing of the lines of longitude as is the case nearer to the poles, such as in the Barents Sea. It was necessary to use the smoothing base ‘cc’ (cyclic cubic regression spline; [51]) for the day of year variable as this enabled the beginning and the end of adjoining years to be linked. Salinity was removed from the final models as it did not significantly help to explain the variance in CPUE or improve the environmental model. Month was also not included in the final models as day of the year performed better in the models.

**Table 1:** CPUE and Prey GAM families and variables. Each of the three prey models were produced for the prey types euphausiids, cod, capelin, herring and for empty stomachs.

Name	Family	Variables
Temporal CPUE GAM	negative binomial	s(latitude, longitude, bs = "sos") + s(year) + s(dayofyear, bs = "cc")
Environmental CPUE GAM	negative binomial	s(latitude, longitude, bs = "sos") + s(surfacetemp, bottomtemp, bs = "ts") + s(depth)
Spatio-temporal CPUE GAM	negative binomial	te(longitude, latitude, yr, bs = "ts")
Temporal Prey GAM	Quasi binomial	s(latitude, longitude, bs = "sos") + s(year) + s(dayofyear, bs = "cc")
Environmental Prey GAM	Quasi binomial	s(latitude, longitude, bs = "sos") + s(surfacetemp, bottomtemp, bs = "ts") + s(depth)
Spatio-temporal Prey GAM	Quasi binomial	te(longitude, latitude, yr, bs = "ts")

Cod stomach content data from the DAPSTOM database for stations in the Barents and Norwegian Seas were combined with CPUE data and temporal, environmental and spatial variables for the same research cruises. Stomach content data were available for 12 years (1930 and 1949–1959), 992 stations, comprising 18,006 cod stomachs in total. The most frequently occurring stomach record (21.4% overall) was ‘empty’. Of the cod that had prey in their stomachs, the most frequent prey types were euphausiids (krill; 21.3%) and capelin (5.6%). Other important prey types included in further analyses were herring (2.3%) and cod (cannibalism; 3.0%), based on the reported importance of these prey types in previous studies [44, 54] and on frequency of occurrence in the DAPSTOM dataset. Frequency of occurrence of these five stomach content categories (hereafter ‘prey’) was converted to a proportion of occurrence at each station and used to develop GAM models exploring the influence of temporal, environmental and spatio-temporal variables on prey choice. The quasibinomial family was used for the frequency of occurrence or absence of each prey type at a station because prey choices were not necessarily independent for each fish at a station; we assumed that if one fish was eating a certain prey species e.g. capelin, it was likely that the other fish in the catch at that station would also be eating that prey. Hyperiid amphipods, the prawn *Pandalus borealis*, the ctenophore *Beroë sp*. and pteropod mollusc *Limacina helicina* were occasionally important prey types at certain localities and times of year, but due to their relative rarity were not included in statistical analyses. Similar to the GAMs for CPUE, salinity was removed from the final GAMs for stomach content as it did not improve explanations of prey frequency; likewise day of year was used rather than month (variables, splines and smooths in Table 1).

## Results

**Fig 2:**
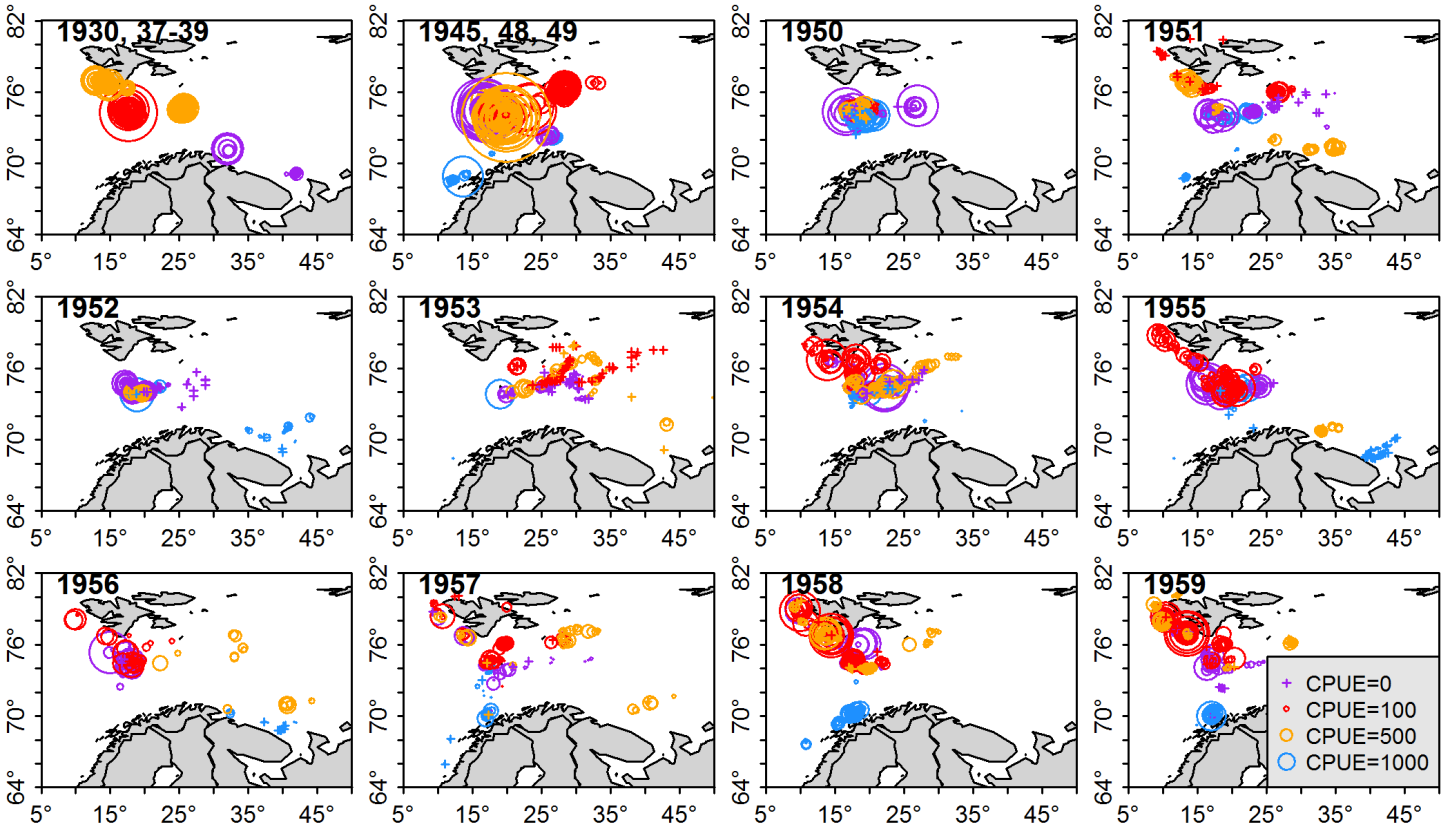
Cod CPUE at each station from 1930–1959. Crosses represent stations where no cod were caught. Size of the circles represent CPUE. Blue = winter, purple = spring, red = summer, orange = autumn.

**Fig 3:**
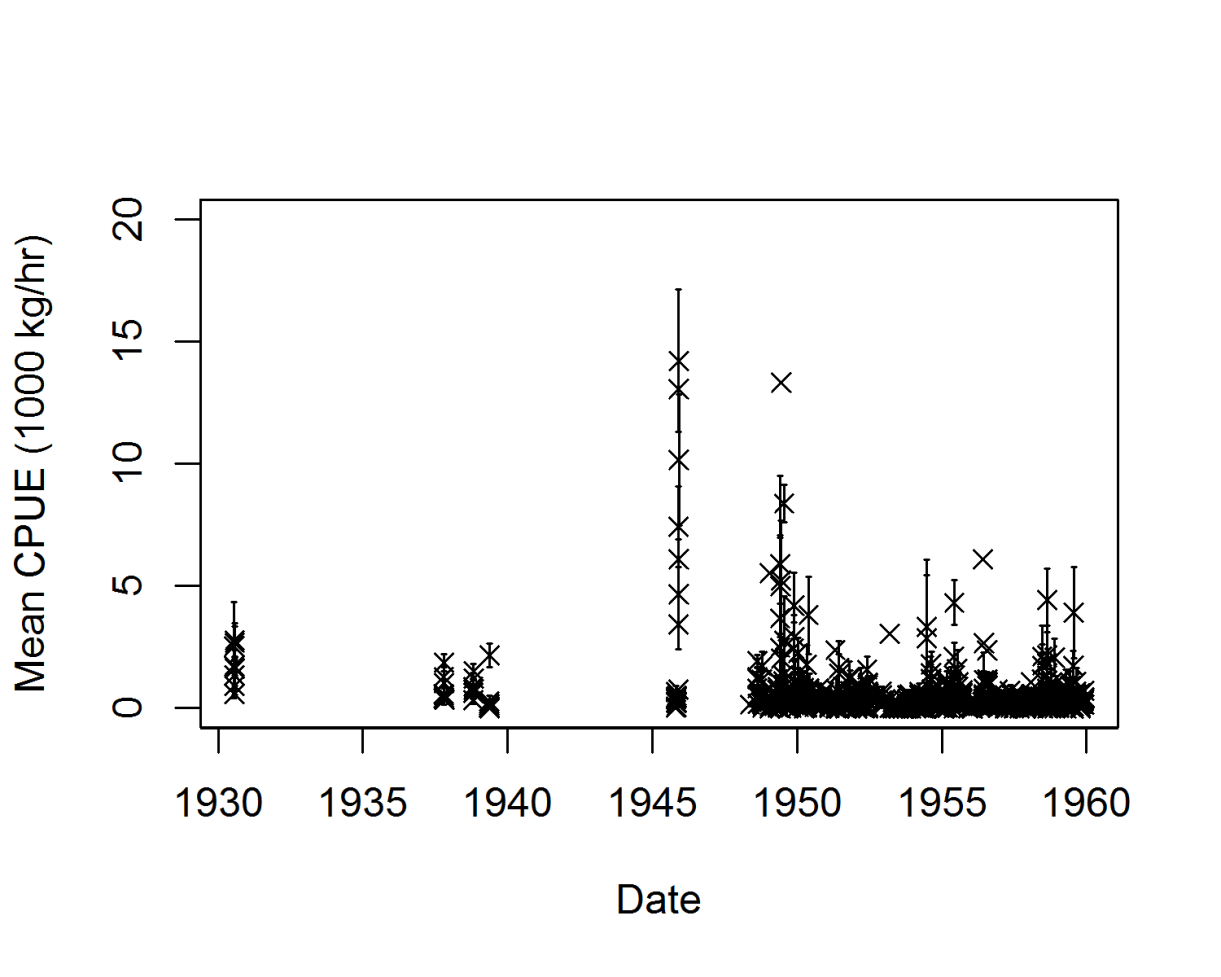
Long-term change in cod CPUE (kg/hr) averaged by day (±SE) for all stations combined. Note the large number of zero and near-zero CPUE values, but also very high CPUE values in some years.

Cod CPUE varied considerably on an annual, seasonal and spatial basis. The largest overall CPUE was in the 1930–1940s (Fig 2). Directly south of Svalbard, where cod were typically caught during spring, summer and autumn, the highest absolute catches were recorded in the 1930–1940s, 1950, 1954 and 1955, while to the west of the archipelago the highest catches were recorded later in the time series, in 1958 and 1959. Around the coast of Norway, close to the main cod spawning grounds around the Lofoten Islands where cod were sampled during winter, the highest catches were in the 1940s, and also in 1958 and 1959, with a high frequency of low or zero CPUE records at stations in 1951 and 1952. When all stations were combined, there were peaks in CPUE from 1945–1949, followed by a sharp decline in CPUE in the early 1950s (Fig 3). This was followed by a slight increase through the mid-1950s and decrease again to the end of the decade. Typical of any fish survey, there was a large amount of variability in CPUE data, with a large number of zero values, but also many very high CPUE values (up to several thousand kg/hr, especially during the late 1940s). A seasonal trend in CPUE was clearly evident from the data, with highest CPUE, and most variation in catch occurring during the summer months (June to August) and in the winter (November and December).

**Fig 4:**
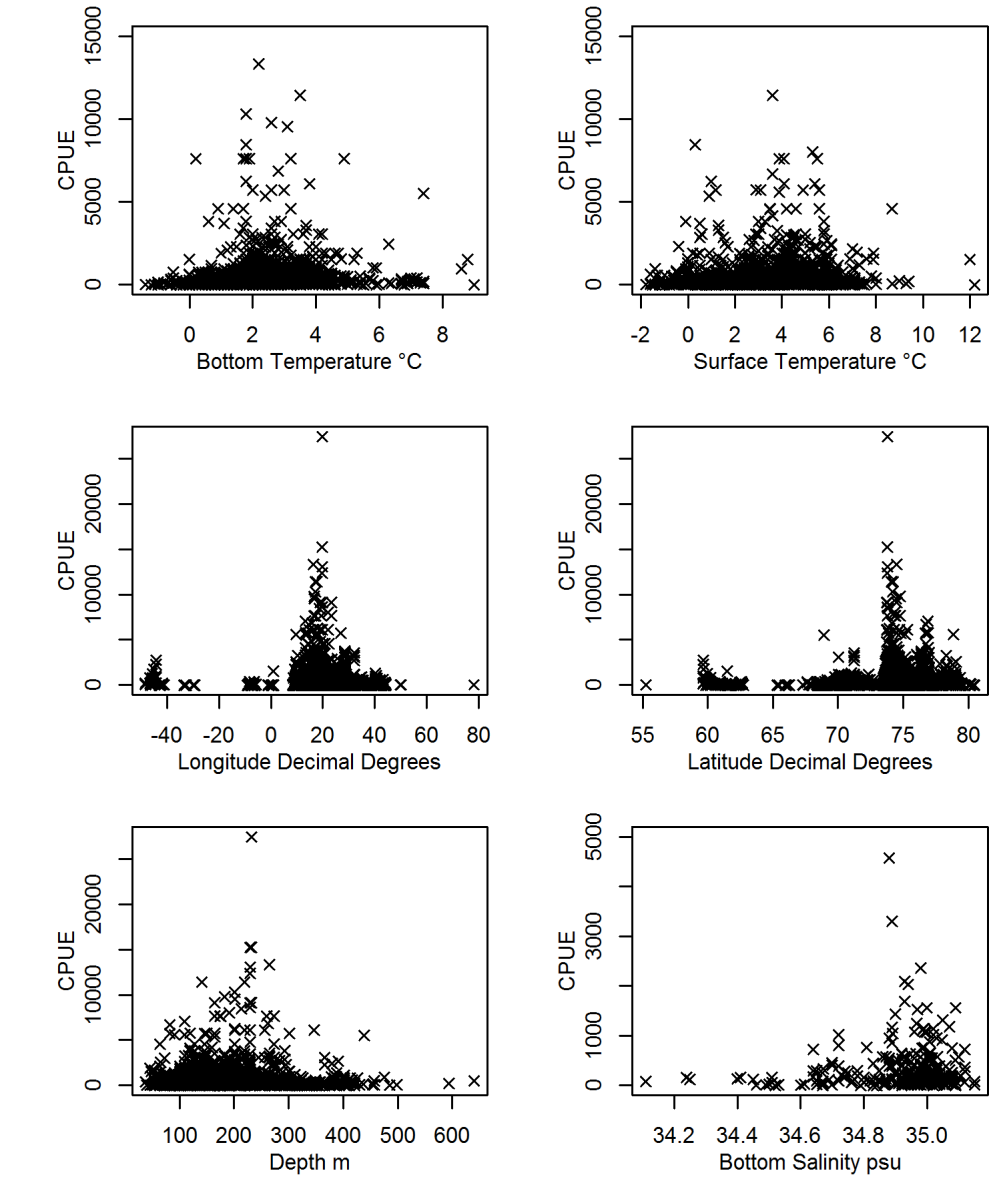
Relationship between CPUE and spatial and environmental variables.

Environmental variables and spatial parameters mirrored trends in cod catches, with the highest CPUE seen when bottom temperatures were 2–4°C and surface temperatures 0–6°C (Fig 4a and 4b). Cod CPUE was greatest from 15–45° E longitude, and 73–77° N latitude (Fig 4c and 4d), from 150–250 m depth (Fig 4e) and with bottom salinities in the range 34.8–35.2 psu (Fig 4f). Drivers for changes in cod CPUE were explored using GAMs. The optimal models found that longitude, latitude, year, day of year, bottom and surface temperature, and depth were important in explaining cod CPUE (Table 2). Deviance explained by each model was 25–29%, suggesting that other factors and natural stochasticity not captured by the models were also influencing CPUE.

**Table 2:** Summary results from GAMs developed to predict cod CPUE. Theta (*θ*) measures dispersion of the negative binomial distribution. df = degrees of freedom

Name	Adj. R-sq	θ	df	Deviance explained (%)	n	Significant
Temporal CPUE GAM	0.23	0.389	63.5	25.1	2741	All
Environmental CPUE GAM	0.06	0.692	69.26	24.9	1218	All
Spatio-temporal CPUE GAM	0.10	0.407	121.1	28.6	2741	All

The environmental model identified that surface temperature was less reliable at explaining CPUE than bottom temperature, with highest CPUE predicted at both high and low surface temperatures. Depth was a good explanatory variable for CPUE between 100–300 m, although with fewer data points at depths less than 100 m and more than 300 m, the model is less reliable at shallower and greater depths. The temporal model supported initial visual analysis of the data (Fig 2), describing high CPUE from 1930 to late 1940s followed by a decline in the early 1950s and a slight increase later in the 1950s. The temporal model suggested variation in catches throughout the year, with highest CPUE in the summer and winter months and lowest CPUE in the spring months. Development of the spatio-temporal model found latitude and longitude, along with time, were valuable descriptors of CPUE.

**Fig 5:**
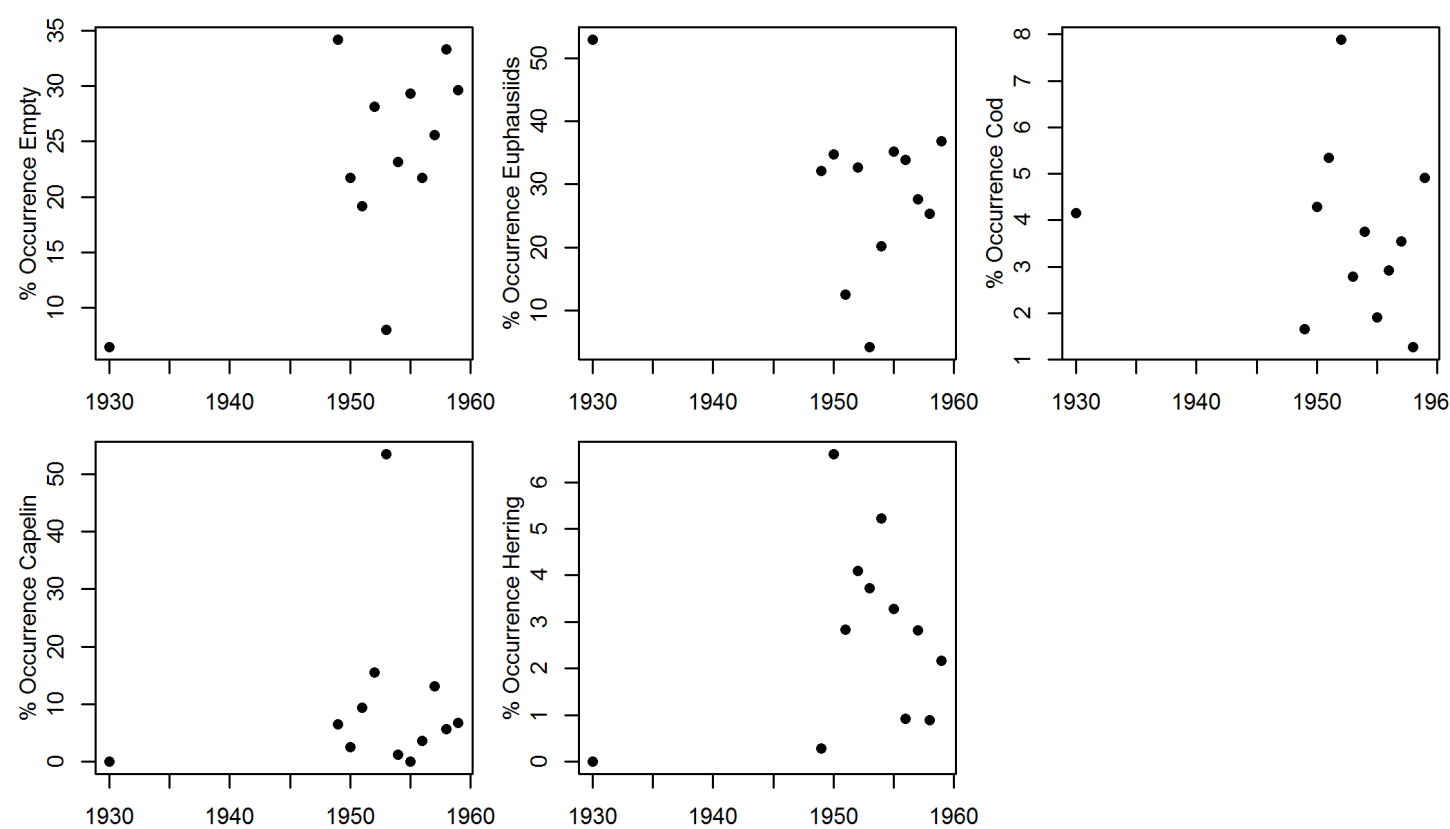
Annual mean percentage occurrence of the five major stomach content categories.

There was substantial interannual variation in prey items identified in cod stomachs from 1930–1959 (Fig 5). Capelin was particularly important in the diet of cod in 1949, 1951, 1952, 1953 and 1957, dominating the diet in 1953. The importance of crustaceans such as euphausiids (but also hyperiid amphipods) was highly variable, contributing a large proportion of prey items in most years except 1951, 1954 and 1956. Cod cannibalism was also variable, with the highest proportions of cod in the diet in 1952 and 1957. Herring occurrence in cod stomachs was low overall in comparison to capelin, with the highest frequencies in 1950 and 1954. The number of empty stomachs and the presence of euphausiids, was particularly low in 1953, when capelin made up the greatest proportion of cod diets.

The GAMs found that spatial, temporal and environmental variables were all associated with cod diet. Longitude, latitude, year, day of year, bottom and surface temperatures, and depth were all valuable descriptors of cod diet (Table 3). Models predicted up to 89% of the variation in occurrence of items in the diet, indicating that these factors can dominate prey choice. Highest explanatory capability was for capelin and herring, suggesting spatio-temporal and environmental drivers, while small cod and euphausiids, and empty stomachs were explained less well by these variables (deviance explained = 26–54%). All variables were significant in all models except for depth in the environmental model for cod as prey.

**Table 3:** Summary results from GAMs developed to predict frequencies of prey in cod diet. df = degrees of freedom

Prey	Model	Adj. R-sq	Total df	Deviance Explained (%)	n	Significant (P < 0.05)
Euphausiid	Environmental GAM	0.44	43.44	43.3	691	All
	Temporal GAM	0.55	55.58	52.7	1025	All
	Spatio-temporal GAM	0.21	44.22	25.5	1025	All
Capelin	Environmental GAM	0.89	82.42	88.4	691	All (depth P = 0.03)
	Temporal GAM	0.91	64.37	89.6	1025	All
	Spatio-temporal GAM	0.89	98.25	89.1	1025	All
Cod	Environmental GAM	0.23	52.97	33.5	691	Not depth
	Temporal GAM	0.40	41.55	42.7	1025	All
	Spatio-temporal GAM	0.39	96.22	44.5	1025	All
Herring	Environmental GAM	0.65	82.76	68.9	691	All
	Temporal GAM	0.63	66.3	71.9	1025	All
	Spatio-temporal GAM	0.56	120	71.1	1025	All
Empty	Environmental GAM	0.45	48.23	45.2	691	All
	Temporal GAM	0.56	53.96	54.1	1025	All
	Spatio-temporal GAM	0.45	80.69	45.7	1025	All

## Discussion

We used previously unavailable analytical techniques to investigate underlying variables responsible for historical changes in the Barents Sea food web. Our analysis improves the knowledge base of the role of climate on the ecology of this region, and demonstrates the importance of rescuing historical datasets, even if incomplete. For example, spatial distribution in catches should be treated with some caution because the location of the sampling stations was not based on a contemporary rigorous statistical survey design; to an extent this could have affected our results on historical cod diets. In addition, because catch weights are based on assumptions of averages (both basket weights and individual fish weights) rather than absolute values, these weights should be considered as relative rather than exact. Nevertheless, analysis of this dataset shows that environmental, spatial and temporal variables were all important descriptors of prey items consumed by cod in the Barents Sea, ultimately influencing catches of cod in the region. This dataset represents a period of time when cod stocks were initially very large and subsequently declined. Catches in surveys were higher in the 1930–1940s compared to the 1950s. Catches were more variable in space and time across the Barents Sea in the 1950s. Lowest predictive power was during the 1930s and 1940s due to a paucity of data; the result of fewer cruises. Previous work has shown that CPUE was higher from 1925–1960 when compared with cooler periods before or since, coinciding with high cod recruitment [55]. The low CPUE observed in spring is likely to be a result of cod, after their return from the winter spawning grounds, dispersing widely to commence feeding.

Cod diet varied significantly between years, with capelin and herring being strongly influenced by environmental variables. A dominance of capelin in the diet was seen in 1953, a cold year, which tends to favour capelin abundance [56]; in this year, few empty stomachs were recorded and there were low proportions of cod and euphausiids in the diet. This peak supports the importance of capelin as a food source for cod over other prey species when available. Analysis of cod stomachs sampled further east during Russian surveys in the 1930s found that haddock *Melanogrammus aeglefinus* and capelin were the preferred prey items, and that euphausiids were eaten only when fish were unavailable [44]. A reported decline in capelin from 1958–1960 [57] coincides with the reduction of capelin in cod stomachs in 1958 in this study. Euphausiid numbers are also affected by climate, which explained 30–60% of the variation in their abundance in one study using stomach data from 1952–2009 [18]. Analysis of more recent stomach contents records (from 1984–2006) similarly found that capelin was the most important prey item by weight (at 30%) [45]. These data have also been used to provide calculations of total prey consumption and energetics [45], to which this newly available data for earlier decades could make a valuable contribution. Cod cannibalism is known to be widespread, and Russian and Norwegian cod stomach content analyses have shown that cod cannibalism is negatively correlated with capelin abundance and varies spatially [58], also found here.

The work here largely confirms analysis from recent decades which have provided a better understanding of impacts of climate on annual movements and distributional shifts in cod. For example, climate variability influences Barents Sea cod distribution and causes a northward distribution shift in warmer years; in the 1920s and 1930s it is thought that cod, haddock and herring *expanded* farther north in the north Atlantic, whereas the range of capelin and polar cod was *shifted* to the north [35]. Water temperature during the first year of life is also a good descriptor of strong year classes; for example weak year classes from 1979 to 1982 coincided with cold years, while strong year classes from 1983–1984 in Barents Sea cod were associated with warm periods [14, 59]. Our analyses of data from preceding decades support the findings of colleagues [37]; that temperature and depth affect cod distribution and abundance. The 2–3°C temperature range occupied by cod in the Barents Sea is considered optimal for this stock [56], corroborating our findings that catches were highest at 2–4°C. Scientists on board the *RV Ernest Holt* reported finding the largest stock at the boundary of the West Spitsbergen Current (Atlantic water) and the Bear Island Current (Arctic water), with cod being particularly concentrated where these two waters mixed [48]. In parallel with cod, the distribution of capelin is also affected by sea temperature, with the population moving north and east in warmer years [35, 57].

The Atlantic Multidecadal Oscillation (AMO), derived from de-trended north Atlantic-wide sea surface temperatures, is important in characterising multi-decadal climate variability and correlates with fish production and changes to spawning sites [15]. Barents Sea warming followed by cooling during the period of this study (1930–1959), illustrates changing phases of the AMO, which has a periodicity of 60–80 years [60]. Thus, effects of changes in Barents Sea temperature and hydrography are the likely cause of changes in the ecosystem [35]. In more recent years there is evidence of changing climatic conditions and fisheries management affecting the ecology of the Barents Sea and the abundance of cod [17]. Zooplankton and fish populations have shifted poleward [15], and since 2003 cod have been spawning further north along the Norwegian coast than any time in the previous 40 years [31]. Inclusion of data from this study in analyses with Norwegian and Russian datasets from more recent decades would allow long term variation in fishing mortality, cod abundance, diets and hydrography to be considered in a context of climate variability. Suggestions for future analyses include testing for a time lag between temperature in previous years and subsequent catches, including fishing intensity information where available, and exploring additional data on prey availability, such as spatio-temporal variability in prey abundance [61–62].

Since the Barents Sea has been fished by many nations, but is highly sensitive to climate change, these data can help inform future management of cod stocks. Understanding the influence of climatic conditions on past trends in cod abundance informs predictions of stocks in the future, but predicting climate change effects in the Barents Sea is non-trivial since climate variability is modulated by the North Atlantic Oscillation (NAO) and AMO as well as potential anthropogenic climate change. This newly available time series on cod abundance and prey provides insight into previous patterns of change in the Barents Sea ecosystem, which are relevant to future change. Climate models for the Barents Sea region predict a 5°C air temperature rise by the end of the century [63], a 1–2°C sea temperature rise, and potential changes to ocean currents and water masses [64]. Under present temperature conditions the population has already expanded northwards, but it could continue eastwards and migrate for a longer period of each year [17], depending upon depth limitations of cod. Simulations of climate change effects suggest that capelin will also move north-eastwards in the Barents Sea in the future [65], and indeed they have moved in this direction in recent years [66]. Simulations predict that Atlantic zooplankton production will increase 20% by 2059, while Barents Sea zooplankton may decrease [67]. Species responses to climate change, and resulting movement of prey are likely to affect cod survival and distributions, with ecosystem-wide implications if phenological changes to spawning cause mis-matches [1, 68].

Our study demonstrates the value of using historical datasets that, although lacking contemporary statistical rigour and consistency of survey design, provide valuable knowledge on abundance, spatial distributions, prey selectivity and underlying climatic drivers. At a global scale, long-term spatio-temporal data are highly patchy, yet are crucial for understanding how climate change can affect marine ecosystems. With further effort, including digitising UK data from 1960–1976 and integrating Russian and Norwegian data, time series of distribution and diets of Barents Sea cod from 1930 to the present day are achievable, which could then be used in climate impact projections. These datasets are made possible by international collaboration and substantial investment in digitising the wealth of historical logbooks kept by marine scientists, fishermen and naturalists of bygone eras. Extensive time series are invaluable for understanding the influences of cyclical and directional climate change on fish stocks.
